# IL-1 Family Antagonists in Mouse and Human Skin Inflammation

**DOI:** 10.3389/fimmu.2021.652846

**Published:** 2021-03-16

**Authors:** Praxedis Martin, Jérémie D. Goldstein, Loïc Mermoud, Alejandro Diaz-Barreiro, Gaby Palmer

**Affiliations:** ^1^Division of Rheumatology, Department of Medicine, Faculty of Medicine, University of Geneva, Geneva, Switzerland; ^2^Department of Pathology and Immunology, Faculty of Medicine, University of Geneva, Geneva, Switzerland

**Keywords:** atopic dermatitis, cytokine, inflammation, interleukin-1, psoriasis, skin

## Abstract

Interleukin (IL)-1 family cytokines initiate inflammatory responses, and shape innate and adaptive immunity. They play important roles in host defense, but excessive immune activation can also lead to the development of chronic inflammatory diseases. Dysregulated IL-1 family signaling is observed in a variety of skin disorders. In particular, IL-1 family cytokines have been linked to the pathogenesis of psoriasis and atopic dermatitis. The biological activity of pro-inflammatory IL-1 family agonists is controlled by the natural receptor antagonists IL-1Ra and IL-36Ra, as well as by the regulatory cytokines IL-37 and IL-38. These four anti-inflammatory IL-1 family members are constitutively and highly expressed at steady state in the epidermis, where keratinocytes are a major producing cell type. In this review, we provide an overview of the current knowledge concerning their regulatory roles in skin biology and inflammation and their therapeutic potential in human inflammatory skin diseases. We further highlight some common misunderstandings and less well-known observations, which persist in the field despite recent extensive interest for these cytokines.

## Introduction

Inflammation is a double-edged sword. On one hand inflammation is required for host defense in response to invading pathogens, toxic compounds or endogenous harmful signals. On the other hand a failure of the body to stop this response will result in tissue or organ damage.

The members of the interleukin (IL)-1 cytokine and IL-1 receptor (IL-1R) families play a key role in the initiation and regulation of inflammatory responses to both infectious and sterile triggers. The biological activity of pro-inflammatory IL-1 family cytokines is controlled at the level of their production and maturation, as well as by natural receptor antagonists and regulators belonging to the IL-1 cytokine family, and by decoy or inhibitory receptors belonging to the IL-1 receptor family. A dysregulation in the balance between pro- and anti-inflammatory components of the IL-1 system may lead to the development of chronic inflammatory pathologies in various tissues, including the skin.

This review summarizes recent advances in the understanding of the biology of anti-inflammatory members of the IL-1 cytokine family, IL-1R antagonist (IL-1Ra), IL-36Ra, IL-37 and IL-38, and of their role in the control of inflammatory responses in human and mouse skin.

## The Skin

### Structure and Physiological Immune Function of Human Skin

The skin, as the outermost organ of the body, is the first line of defense shielding internal tissues from threats of the outside world. The skin immune system is adapted to meet these specific requirements and includes a number of cell types with sentinel functions. In addition, the physical barrier formed by epithelial cells, the specific chemical composition of the outer skin layers, and beneficial commensal microbiota at the skin surface contribute to its protective properties [reviewed in Eyerich et al. (1)].

**Figure 1:**
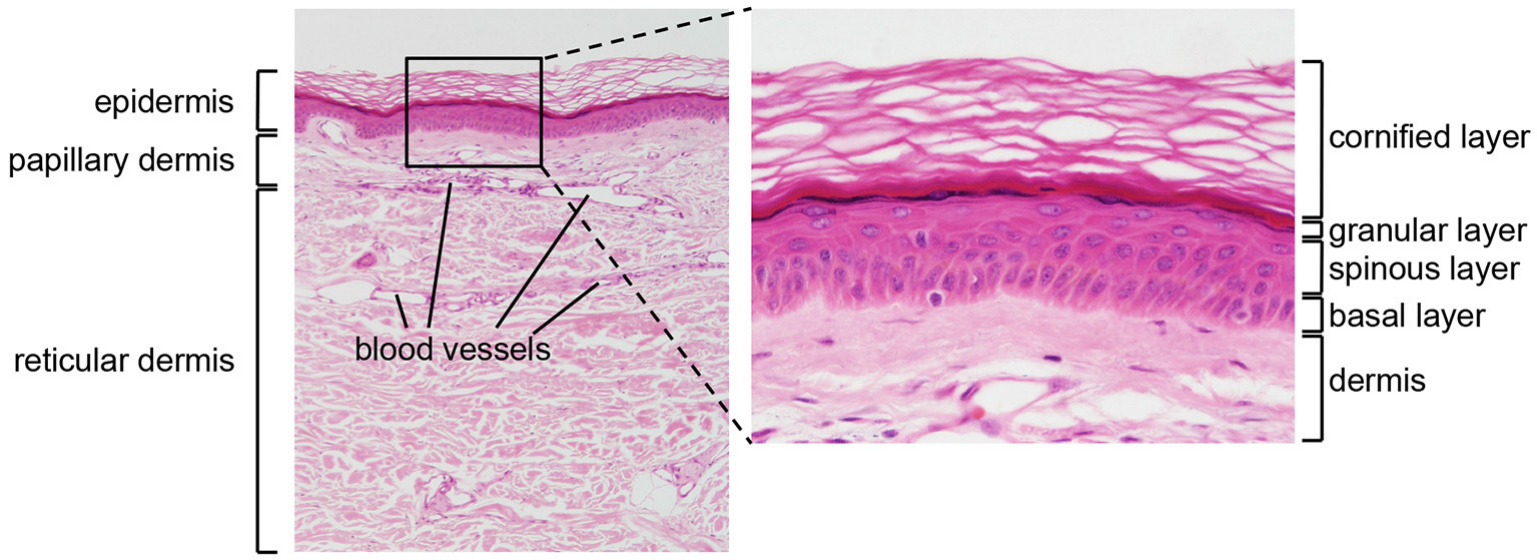
Structure of human skin. HE stained section of normal human skin at ×10 (left panel) and ×40 (right panel) original magnification. Epidermis, papillary (upper), and reticular (lower) dermis are shown (left panel). The epidermis contains keratinocytes arranged from bottom to top in four typical layers: basal layer, spinous layer, granular layer, and cornified layer (right panel). Photomicrography image credit: Lutz Slomianka 1998–2009, Blue Histology (http://www.lab.anhb.uwa.edu.au/mb140/).

The skin is divided into two main compartments, the epidermis and the dermis, which differ in their structure and functions (Figure 1).

The epidermis is the external layer of the skin and constitutes the interface with the environment. It is formed by a cell dense stratified epithelium. The human epidermis contains keratinocytes arranged in four successive layers defined as: a basal layer (or *stratum basale*, SB), a spinous layer (*stratum spinosum*, SS), a granular layer (*stratum granulosum* SG), and a cornified layer (*stratum corneum*, SC). Each layer is characterized by specific morphological and biochemical features related to the differentiation state of the keratinocytes, which increases from the SB to the SC. The SB contains a single layer of cuboidal, proliferating progenitor cells attached to a basement membrane. Post-mitotic keratinocytes first move to the SS, and then to the SG, while expressing successive differentiation markers (2). Finally, the SC contains terminally differentiated flattened, enucleated keratinocytes, surrounded by a rigid structure, the cornified envelope, which consists of cross-linked insoluble proteins covalently bound to lamellar lipid layers. The SC provides most of the physical barrier of the skin and is responsible for its biomechanical stability and hydrophobic properties. In addition, the lower epidermal layers contribute to barrier function through different types of intercellular junctions, which ensure mechanical cohesion (3).

The epidermis contains interspersed specialized immune cells, in particular Langerhans cells (LC) and resident T lymphocytes. LC are tissue-resident macrophages sharing many functional features with dendritic cells (DC) (4). Indeed, LC function as antigen-presenting cells and migrate to lymph nodes, even at homeostasis, to present epidermal antigens to antigen-specific T cells. Their role in shaping local and systemic immunity is complex, as they can coordinate induction of adaptive immune responses or tolerance, depending on the context (5). In human skin, epidermal-resident lymphocytes are mainly TCRα/β CD8^+^ tissue-resident memory T (TRM) cells, which are believed to be crucial for local immunity and recall responses (6). Finally, the keratinocytes themselves play a key role as early detectors of microbial danger signals. In response, they secrete chemokines and cytokines, as well as antimicrobial peptides (AMP), which, in addition to their direct antimicrobial properties, also act as chemoattractants for immune cells (7).

The dermis is a layer of connective tissue lying beneath the epidermis, from which it is separated by a basement membrane. It contains sparse dermal fibroblasts involved in the synthesis of a collagen-rich extracellular matrix, which confers tensile strength to the skin. Dermal fibroblasts also express a broad repertoire of inflammatory mediators, which can be produced in large amounts. In addition, the dermis contains a variety of immune cells with specialized functions, including different subsets of macrophages and conventional (c)DCs and mast cells (8). The dominant dermal-resident lymphocyte subset corresponds to TCRα/β CD4^+^ TRM cells (9). Finally, type 1, 2, and 3 innate lymphoid cells (ILC) are also found in healthy human dermis (10).

### Skin Inflammation

The skin constitutes a complex environment, with constant interaction of immune and stromal cell types, which are all capable of performing immune functions. Maintenance of skin immune homeostasis requires a well-harmonized equilibrium between each of these actors, and dysregulation may lead to the development of inflammatory skin diseases, including psoriasis and atopic dermatitis (AD) (11, 12).

Psoriasis is typically characterized by the presence of well-demarcated erythematous (exhibiting abnormal redness due to blood accumulation) plaques covered by silvery lamellar scales (13), while AD is characterized by recurrent eczematous lesions and intense itch, and can evolve into chronic dermatitis with lichenification (area of hard, thickened skin) of the epidermis (14). Both pathologies are associated with hyper-proliferation and altered differentiation of keratinocytes, leading to acanthosis (increased thickening of the epidermis), and parakeratosis (abnormal epidermal keratinization characterized by loss of the SG and retention of nuclei in keratinocytes of the SC) (13, 14). Disease may be initiated by an abnormal epidermal response to stress such as *Staphylococcus aureus* colonization, chemical irritants or sunburn, for psoriasis (13), or by an alteration in keratinocyte function, facilitated by mutations of sensitivity genes, such as filaggrin, and inducing an increase in permeability and altered integrity of the epidermal barrier, for AD (14). In both pathologies, early pathogenic events include innate immune responses, which trigger infiltration of specific cell subtypes and establishment of a susceptible environment, before adaptive immunity, and notably T cells favor the transition toward chronicity of the disease (14, 15).

An important difference between psoriasis and AD lies with the cytokine axes mediating the pathology: the Th17 axis for psoriasis, and the Th2 axis for AD (14, 16, 17). Even before T cell involvement, keratinocytes polarize the immune response by producing Th17 or Th2 promoting cytokines, or by controlling their production by other skin cells, such as ILC3 (16) or ILC2 (17).

### Differences Between Mouse and Human Skin

Mouse models are essential to investigate the complex mechanisms occurring both locally and systemically in the context of inflammatory skin diseases. However, the structure and cellular composition of mouse skin present several important differences with human skin. Indeed, epidermis and dermis are thicker in human, with several cell layers in particular in the epidermal SS and SG, against only one in murine epidermis. Human skin also presents rete ridges (downward projection of the epidermis between the dermal papillae) included in the dermis, and large areas of inter-follicular skin with few hair follicles. On the contrary, mice have numerous and densely packed hair follicles (11). In addition, while human skin contains sweat glands at variable density throughout the body, these are found only in footpads in the mouse (18).

Regarding skin immune cell populations, 90% of mouse epidermal T cells at homeostasis represent a subset of Vγ5^+^ TCRγδ^+^ dendritic epidermal T cells, which is absent from human skin (11, 12, 19). Human skin in contrast contains important numbers of LCs and CD8^+^ TRM cells, which is not the case in mice (11).

Functionally, immune mechanisms at homeostasis and during inflammation are also different, especially regarding the involvement of T cells. For instance, human but not murine LCs express CD1a (20), making them capable of presenting microbial non-peptide antigens to T cells (12, 21). During Aldara [5% imiquimod (IMQ)]-induced skin inflammation, one of the most widely used mouse models of psoriasis (22), production of IL-17, the center molecule of the Th17 axis, is mediated mostly by Vγ4^+^ TCRγδ^+^ dermal T cells (23–26). On the contrary, in human psoriasis, skin T cells are dominated by TCRαβ^+^ T cell subsets, which are the main producers of IL-17, while TCRγδ^+^ T cells represent only 1% of the T cell population (27).

Finally, early production of type I IFN by plasmacytoid (p)DCs is essential for the development of acute human psoriasis (15, 28). However, in Aldara (5% IMQ)-induced murine skin inflammation, type I IFN is not detected during the course of the disease, even at early times (29). Thus, although mouse models are precious to understand the mechanisms of skin inflammation, interspecies differences need to be taken into account for correct interpretation of experimental results.

## IL-1 Family Cytokines, Receptors and Signaling

The IL-1 system comprises 11 cytokines, 10 structurally related receptors and the receptor-like soluble protein IL-18 binding protein (IL-18BP).

### IL-1 Family of Cytokines

The cytokines of the IL-1 family are IL-1α, IL-1β, IL-1Ra, IL-18, IL-33, IL-36α, IL-36β, IL-36γ, IL-36Ra, IL-37, and IL-38. They are encoded by genes located in the IL-1 gene cluster on chromosome 2, both in human and mouse, except for IL-18 (human: chromosome 11; mouse: chromosome 9) and IL-33 (human: chromosome 9; mouse: chromosome 19).

All these proteins function as extracellular cytokines, which exert either pro-inflammatory (IL-1α, IL-1β, IL-18, IL-33, IL-36α, IL-36β, IL-36γ) or anti-inflammatory (IL-1Ra, IL-36Ra, IL-37, IL-38) effects. Interestingly, except for IL-1Ra, cytokines of the IL-1 family lack an N-terminal signal peptide and are not secreted through the classical ER-Golgi pathway. However, they can all be released from their producing cells by different, mostly unknown mechanisms, and exert extracellular biological functions by binding to specific, membrane-anchored, cell surface IL-1 family receptors [for review Carta et al. (30)]. In addition, for some of the members (IL-1α, IL-1Ra, IL-33, IL-37), intracellular effects have been described (31–38).

Most IL-1 family cytokines are expressed as precursors, which can be proteolytically cleaved by different proteases into mature forms (39). Pro-IL-1β and pro-IL-18 are biologically inert and require processing by caspase-1, upon inflammasome activation, to generate mature bioactive forms, which are then released from the cells. In addition to caspase-1, pro-IL-1β, and pro-IL-18 can be matured by proteinase 3, elastase, chymase, or granzyme B (40–43). Unlike pro-IL-1β and pro-IL-18, pro-IL-1α, pro-IL-33, and pro-IL-37 are biologically active. However, their activity is markedly enhanced upon cleavage and maturation by specific proteases (44–46). IL-36α, IL-36β, IL-36γ, or IL-36Ra, respectively, require N-terminal truncation by the neutrophil granule-derived proteases cathepsin G, proteinase 3 or elastase, or removal of the N-terminal methionine to reach their full biological potential (47–49). Anti-inflammatory bioactivity of IL-38 has also been proposed to depend on N-terminal maturation, but the mechanism involved is still unknown (50).

Finally, pro-IL-1α and pro-IL-33 contain a nuclear localization sequence in their N-terminal domain and are found in the nucleus of their producing cells, where they exhibit regulatory properties (35, 51, 52). These cytokines are considered to act as alarmins, i.e., constitutively expressed intracellular molecules, which are released after necrotic cell damage to rapidly activate immune cells by binding extracellularly to specific receptors (53).

### IL-1 Family Receptors and Signaling

The IL-1 receptor family consists of 10 structurally related members and includes 4 ligand-binding receptors (IL-1 receptor type 1 (IL-1R1); IL-18Rα; ST2, also termed IL-1R like 1 (IL-1RL1); IL-36R), two receptor accessory proteins (IL-1 receptor accessory protein (IL-1RAP); IL-18 receptor accessory protein [IL-18RAP)], two receptors, which inhibit signaling [IL-1R type 2 (IL-1R2); single Ig domain containing IL-1 receptor-related molecule (SIGIRR), also termed TIR-8] and two orphan receptors with unknown functions (X-linked IL-1R accessory protein like 1 (IL-1RAPL), also termed TIGIRR-2; X-linked IL-1R accessory protein like 2 (IL-1RAPL2), also termed TIGIRR-1). Many of the genes encoding IL-1 receptors map to chromosome 2 in humans and chromosome 1 in mice. The gene coding IL-1RAP maps to chromosome 3 (human) or 16 (mouse), IL-36R to chromosome 2 (mouse), SIGIRR to chromosome 11 (human) or 7 (mouse) and TIGIRR-2 and TIGIRR-1 to the X chromosome (human and mouse).

All receptor family members contain 3 extracellular immunoglobulin (Ig)-like domains and one transmembrane domain, with the exception of SIGIRR, which comprises only one extracellular Ig-like domain (54). Except for IL-1R2, the receptors further share a conserved intracellular Toll/IL-1 receptor (TIR) signaling domain and SIGIRR, TIGIRR-2 and TIGIRR-1 have an additional C-terminal cytoplasmic extension, which is reminiscent of Drosophila Toll (55, 56). Specific ligands for SIGIRR and TIGIRR-1 have not been identified so far, while a recent study suggested IL-38 as a ligand for TIGIRR-2 (50).

An exception and not member of the IL-1 receptor family is IL-18BP, which is a receptor-like soluble protein mapping to chromosome 11 (human) or 7 (mouse). IL-18BP does not share any clear sequence homology with other proteins. It consists of a single Ig-like domain and therefore resembles the extracellular part of IL-1 family receptors. IL-18BP lacks a transmembrane domain and is not bound to the cell surface.

**Figure 2:**
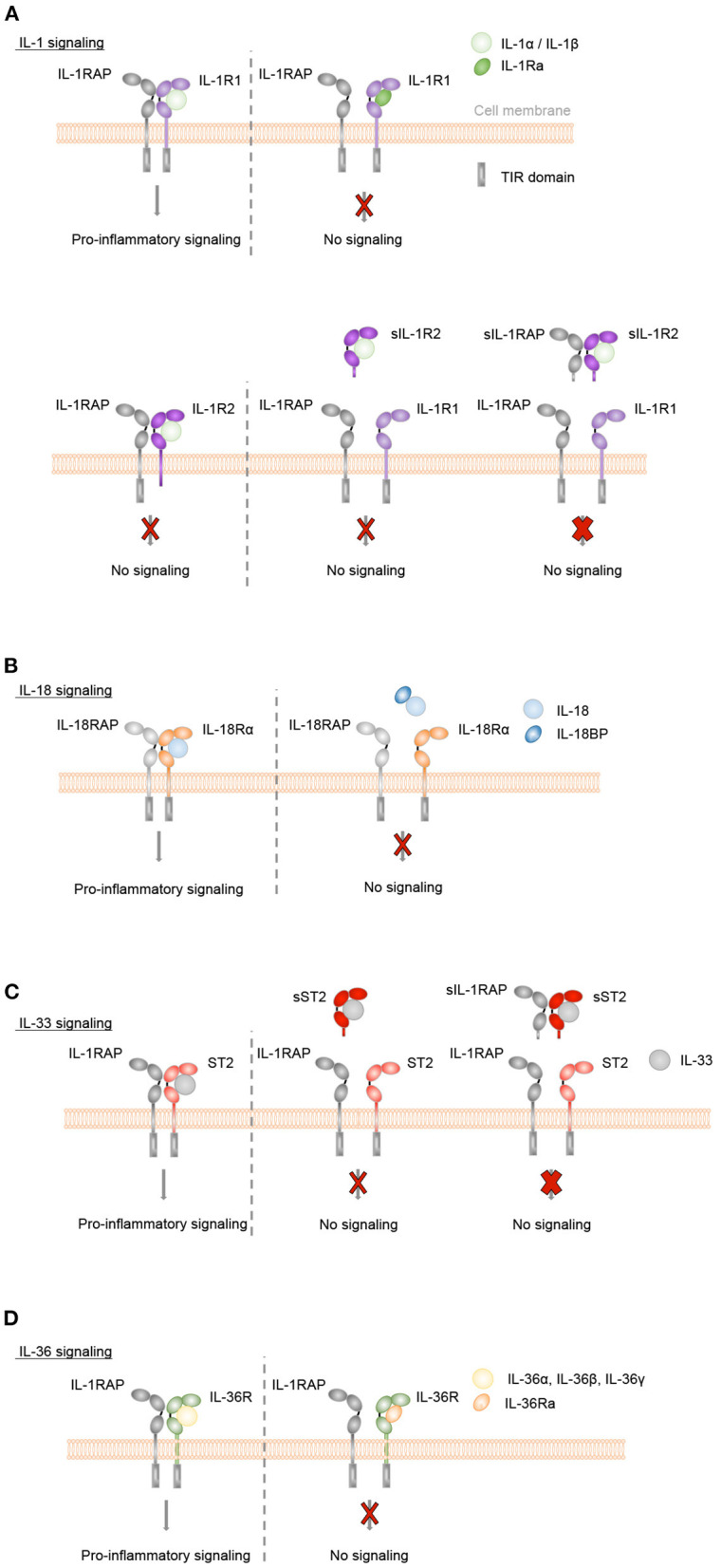
Inflammatory IL-1, IL-18, IL-33, and IL-36 signaling is controlled by natural antagonists and regulatory molecules of the IL-1 family. **(A–D)** Upon binding of IL-1α or IL-1β to IL-1R1, of IL-33 to ST2, of IL-36α, IL-36β or IL-36γ to IL-36R or of IL-18 to IL-18Rα the co-receptors IL-1RAP or IL-18RAP, respectively, are recruited. Signaling is then initiated by the juxtaposition of the cytoplasmic TIR domains, which are present in both the ligand-binding and accessory protein chain. This leads to MyD88 and IRAK binding, activation of NF-κB and mitogen-activated protein kinase (MAPK) pathways and a pro-inflammatory signaling cascade. In order to control these inflammatory responses, different mechanisms exist. **(A+D)** The natural antagonists IL-1Ra or IL-36Ra bind to IL-1R1 or IL-36R, respectively, without recruiting the co-receptor IL-1RAP. Receptor antagonist binding competitively inhibits IL-1 cytokine- or IL-36 cytokine-mediated signaling. **(A)** Membrane-bound and soluble IL-1R2 (sIL-1R2), which lack the intracellular TIR domain, act as decoy receptors and bind to IL-1α or IL-1β with high affinity. In the case of membrane-bound IL-1R2, the IL-1RAP co-receptor is recruited. However, as IL-1R2 lacks the cytoplasmic TIR domain, no signaling cascade is initiated (thin red cross). Soluble IL-1RAP (sIL-1RAP) increases the affinity of sIL-1R2 binding to IL-1 and thus enhances the ability to inhibit IL-1 activity (thick red cross). In addition, IL-1R2 sequesters IL-1RAP thereby blocking IL-1R1/IL-1RAP receptor complex formation. **(B)** IL-18BP prevents the binding of IL-18 to its receptors IL-18Rα and IL-18RAP and acts as a soluble decoy receptor to control excessive IL-18-mediated inflammatory responses. **(C)** IL-33-mediated signaling can be inhibited by the soluble decoy receptor sST2 (thin red cross), with sIL-1RAP further enhancing the ability of sST2 to inhibit the effect of IL-33 (thick red cross).

IL-1α and IL-1β bind specifically to IL-1R1, IL-18 to IL-18Rα, IL-33 to ST2 and IL-36α, IL-36β, and IL-36γ to IL-36R (Figures 2A–D). Upon binding of these cytokines to their specific ligand-binding receptor chain, a receptor accessory protein chain is recruited resulting in the formation of a heterodimeric receptor complex, in which IL-1RAP is the co-receptor for IL-1R1, ST2, and IL-36R, and IL-18RAP for IL-18Rα. Signaling is then initiated by the juxtaposition of the cytoplasmic TIR domains, which are present in both the ligand-binding and accessory protein chain. This leads to the recruitment of the adaptor protein myeloid differentiation factor 88 (MyD88) and IL-1R-associated kinases (IRAK) and further to the activation of NF-κB and mitogen-activated protein kinase (MAPK) pathways, resulting in the expression of various inflammatory genes.

### Antagonists and Other Inhibitory Molecules of the IL-1 System

The potent pro-inflammatory effects of IL-1 family cytokines are tightly controlled by different types of negative regulators.

The naturally occurring antagonistic IL-1 cytokine family members IL-1Ra and IL-36Ra regulate the biological activity of IL-1α and IL-1β or IL-36α, IL-36β, and IL-36γ, respectively. They bind with high affinity and specificity to their respective receptors, IL-1R1 or IL-36R, but do not recruit the accessory protein IL-1RAP. They competitively block the binding of the pro-inflammatory cytokines to the receptors and thus act as classical receptor antagonists (Figures 2A,D). In addition, the IL-1 family cytokines IL-37 and IL-38, which appear to display broad anti-inflammatory properties, are also considered as negative IL-1 family regulators.

The activity of IL-1 family cytokines is further modulated by inhibitory IL-1 family receptors. IL-1R2 can bind IL-1α or IL-1β with high affinity and recruits the IL-1RAP co-receptor. However, IL-1R2 lacks a cytoplasmic TIR domain and is thus incapable of inducing a signaling cascade (57) therefore acting as a decoy receptor. In addition, IL-1-bound IL-1R2 sequesters IL-1RAP and thereby blocks IL-1R1/IL-1RAP receptor complex formation (Figure 2A). In addition to its membrane-anchored form, IL-1R2 is found as a soluble decoy receptor (sIL-1R2), which is shed from the cell surface by proteolytic cleavage (58–62). A soluble form of IL-1RAP (sIL-1RAP) enhances the ability of sIL-1R2 to inhibit IL-1 bioactivity (63).

Bioactive IL-18 is bound with high affinity by IL-18BP, which is constitutively present in high concentrations in the circulation (64). IL-18BP prevents the binding of IL-18 to its receptors IL-18Rα and IL-18RAP and thus controls excessive IL-18-mediated inflammatory responses (65, 66) (Figure 2B).

The biological effects of IL-33 can be controlled by a soluble short isoform of the ST2 receptor (sST2), which is generated by alternative splicing of the ST2 gene (67). sST2 lacks transmembrane and cytoplasmic domains. It binds with high affinity to IL-33 and inhibits the formation of a signaling complex with membrane-bound ST2. Thus, sST2 acts as a soluble decoy receptor to control excessive IL-33-mediated signaling (68). This inhibitory activity of sST2 is further enhanced by the presence of soluble IL-1RAP (sIL-1RAP) (69) (Figure 2C).

**Figure 3:**
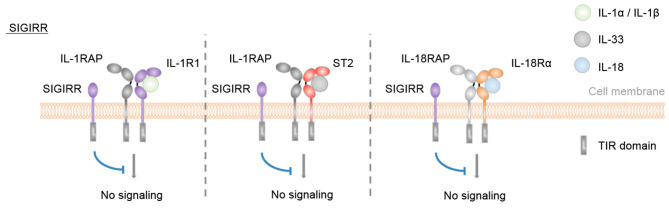
SIGIRR is an inhibitory IL-1 family receptor. No specific ligand for SIGIRR has been described. However, SIGIRR can disrupt IL-1-, IL-33-, and IL-18-mediated signaling by competing for MyD88 and IRAK recruitment.

Finally, SIGIRR is a receptor with negative regulatory functions on IL-1R1, IL-18Rα, ST2, and TLR signaling pathways (54, 70–74) (Figure 3).

In this review, we will focus on the inhibitory IL-1 family cytokines IL-1Ra, IL-36Ra, IL-37, and IL-38, which are all constitutively expressed in human keratinocytes. Their regulatory functions in skin inflammation will be discussed in the following sections.

## Natural Antagonists and Anti-inflammatory IL-1 Family Cytokines in Skin Inflammation

Dysregulated IL-1 family signaling is linked to a variety of skin disorders. The best-studied examples are psoriasis and AD.

In the context of psoriasis, microbial or endogenous danger signals are known to induce IL-36 production by human and mouse keratinocytes (75–78). In the mouse Aldara (5% IMQ) model, IL-36 signaling in keratinocytes is then crucial for disease initiation by mediating the recruitment of neutrophils (24, 79), and controlling the expression of IL-23 at early time points (79). IL-23 in turn promotes IL-17 and IL-22 production by ILC3s, Th17 and TCRγδ^+^ T cells (16) and IL-17 and IL-22 cause keratinocyte hyper-proliferation and altered differentiation, which are typical features of the disease (16, 80, 81). IL-1 signaling plays a complementary role in the pathogenesis of psoriasis but, unlike IL-36, is not fundamental for its induction in mice (24).

Keratinocyte production of IL-33, on the other hand, plays a major role in the initiation of AD. IL-33 notably induces the production of IL-5 and IL-13 by ILC2, which leads to the recruitment of basophils. IL-33 also stimulates basophils to produce IL-4, further enhancing production of IL-5 and IL-13 by ILC2s, and down-regulates the expression of epidermal structural proteins, such as claudin-1 and filaggrin, which aggravates dermatitis [for review Imai (17)].

Anti-inflammatory cytokines of the IL-1 family may thus be expected to play important roles in controlling the pro-inflammatory activities of these different IL-1 family agonists in the skin.

### IL-1Ra

#### IL-1Ra Expression, Activity, and Signaling

Four isoforms of IL-1Ra are produced from the same *IL1RN* gene [gene ID: 3557, human (*IL1RN*; aliases: *IL1F3, IL1RA*); 16181, mouse (*Il1rn*; alias: *Il-1ra*); for review (82)]. The original and best-described isoform is secreted IL-1Ra (sIL-1Ra). It is mainly expressed by activated macrophages, DCs, monocytes, but also by hepatocytes as an acute-phase protein (83). It is synthesized as a pro-protein (pro-sIL-1Ra) containing a cleavable signal peptide and is secreted through the classical ER-Golgi pathway, as a 22-25 kDa glycoprotein (84–88). The three other isoforms lack a signal peptide, are thus considered to stay intracellular and named icIL-1Ra1, icIL-1Ra2, and icIL-1Ra3.

The 18 kDa icIL-1Ra1 isoform is generated by alternative splicing of an icIL-1Ra1-specific first exon, into an internal splice-acceptor site within the exon coding for the signal peptide of sIL-1Ra. The icIL-1Ra1 isoform is predominantly and constitutively expressed by keratinocytes, but is found also in endothelial and myeloid cells (89, 90). The cDNA for a second intracellular isoform (icIL-1Ra2) was cloned from human neutrophils and includes an additional exon located downstream of the first icIL-1Ra1-specific exon. IcIl-1Ra2 has a predicted molecular weight of 25 kDa. However, a corresponding protein has not been described (91). The 16 kDa icIL-1Ra3 is produced by alternative translation initiation from the mRNA encoding sIL-1Ra (92). Despite the lack of a signal peptide, the 3 intracellular isoforms can be passively released by dying cells or actively secreted by leaderless pathways (93–97). Of note, in contrast to the other IL-1 cytokine family members, the IL-1Ra isoforms do not require N-terminal processing to become active.

The major IL-1Ra isoform in mouse and human skin is icIL-1Ra1, which is constitutively expressed in keratinocytes (89, 90, 94, 98–101). Its expression is observed throughout normal mouse epidermis (94, 102), which consists only of 2 or 3 keratinocyte cell layers, so that the identification of different *strata* is difficult. During Aldara (5% IMQ)-induced skin inflammation, expression of icIL-1Ra1 was restricted to keratinocytes of mid and outer layers of the epidermis (94). In humans, whereas some studies suggest icIL-1Ra1 expression in all layers of normal skin (98, 103), icIL-1Ra1 protein expression has been reported to be concentrated in the SG of normal human skin and in the basal-midbasal layers of psoriatic epidermis (99).

In naïve mouse keratinocytes and skin of healthy individuals, the icIL-1Ra1 protein is found in the cytoplasm, whereas Aldara (5% IMQ)-induced inflammation causes presence of the protein additionally in the nucleus of mouse keratinocytes (94, 99). There is currently no explanation for this nuclear localization.

Although the function of sIL-1Ra, which is to compete extracellularly with IL-1α and IL-1β for the binding to IL-1R1 and thereby control IL-1-mediated inflammation (Figure 2A), has been intensively studied, specific functions of icIL-1Ra remain unclear. *In vitro* assays with recombinant icIL-1Ra isoforms demonstrated that they can compete extracellularly for IL-1R1 binding similarly to sIL-1Ra (104, 105). In addition, Irikura et al. demonstrated by the use of genetic epistasis that the activity of all IL-1Ra isoforms depends on the presence of a functional IL-1R1 receptor *in vivo*, suggesting that the only essential function of icIL-1Ra in health and disease is the competitive inhibition of IL-1R1 (106).

Some studies nevertheless reported intracellular roles for icIL-1Ra1 *in vitro* in modulating inflammatory responses, cell proliferation or differentiation in different cell types, including keratinocytes (33, 34, 107–109), but others could not confirm intracellular effects (32, 94, 105, 110).

Concerning the mode of action of icIL-1Ra1 in the skin, extracellular icIL-1Ra1 released by keratinocytes has been proposed to counter-regulate skin inflammation provoked by keratinocyte-derived IL-1α and/or by IL-1β, the latter mainly produced by infiltrating myeloid cells (94, 102, 111). Even though decreased icIL-1Ra1 expression has been detected in lesional psoriatic skin compared to uninvolved psoriatic or normal skin (98, 99), an increased ratio of icIL-1Ra1 to IL-1α, mainly due to the reduction of IL-1α, has been reported in human inflammatory skin diseases, including psoriasis or AD (99, 112, 113). Changes in the icIL-1Ra1/IL-1 ratio in the epidermis may thus reflect a regulatory process occurring in various inflammatory skin conditions.

Taken together, these studies indicate that icIL-1Ra1, which is mainly expressed by keratinocytes, is the major IL-1Ra isoform in both human and mouse skin. In contrast to the well-described role of secreted IL-1Ra, the specific extracellular and/or intracellular function(s) of icIL-1Ra1 remain(s) widely unclear. Nevertheless, icIL-1Ra1 appears to exert anti-inflammatory activity in skin (Table 1) and a dysregulated IL-1 to IL-1Ra ratio may lead to inflammatory skin pathologies (**Figure 5**).

**Table 1:** IL-1 family antagonist expression, activity, and signaling.

**Cytokine**	**Producing cell**	**Target cell**	**Receptor**	**Effect**
IL-1Ra	Keratinocytes (89, 90, 94, 98–101) Infiltrating myeloid cells (89, 94)	Keratinocytes Fibroblasts Myeloid cells	IL-1R1	Inhibition of IL-1 activity (94, 102, 111)
IL-36Ra	Keratinocytes (103, 114–118) Myeloid cells (114, 115, 119, 120)	Keratinocytes Myeloid cells CD4^+^ T cells	IL-36R	Inhibition of IL-36 activity (121–124)
IL-37	Keratinocytes (103, 125–127)	Myeloid cells	IL-18Rα	Broad inhibition of pro-inflammatory signaling (128–132)
			IL-18RAP	Inhibition of IL-18 activity (133)
IL-38	Keratinocytes (103, 117, 118, 124, 134–136)	γδ T cells	TIGIRR-2	Inhibition of IL-17A production (135)
		Myeloid cells	TIGIRR-2 IL-36R?	Broad anti-inflammatory activity (50, 137)
		Keratinocytes	IL-36R?	Inhibition of IL-36γ activity (124)

*For each antagonist, the main producing cell type(s), target cells, receptors, and effects relevant to skin biology are listed. A comprehensive description of biological effects reported for each cytokine can be found in the text*.

#### IL-1Ra in Human Inflammatory Skin Diseases

Polymorphisms in the *IL1RN* gene have been associated with allergic contact dermatitis (138) and psoriasis (139). Furthermore, a life-threatening systemic inflammation with skin and bone involvement has been linked to the deficiency of IL-1Ra (DIRA). The DIRA syndrome is an autosomal, recessive, autoinflammatory disease, which is characterized by neonatal-onset pustular dermatitis (inflammation of the skin that presents with pustular lesions), multifocal aseptic osteomyelitis (inflammation of the bone), periostitis (inflammation of the periosteum, a layer of connective tissue that surrounds bone), leukocytosis, marked elevation of acute-phase reactants such as C-reactive protein and increased *ex vivo* inflammatory cytokine secretion (140, 141). The etiology has been linked either to homozygous mutations in the *IL1RN* gene, which resulted in a truncated IL-1Ra protein that is not secreted (140) or has lost its affinity for the IL-1 receptor (142), or to a 175-kb genomic deletion of chromosome 2q13 that includes *IL1RN* as well as the genes encoding 5 other IL-1-family members, IL-36γ, IL-36α, IL-36β, IL-36Ra, and IL-38 (140, 141). Heterozygous carriers are asymptomatic. These genetic disorders render cells hyper-responsive to IL-1α and IL-1β due to the lack of a functional antagonist.

Children with DIRA responded successfully to daily subcutaneous injection of Anakinra (140, 143). Anakinra is rapidly metabolized and daily injections are thus required to maintain its therapeutic effects. Discontinuation leads to fast relapse of the symptoms. Of note, the DIRA symptoms of patients with the 175-kb genomic deletion including *IL1RN* and five other members of the IL-1 family are more refractory to Anakinra treatment than those of the patients carrying a mutation only in the *IL1RN* gene (140).

Off-label usage of Anakinra has also demonstrated its effectiveness in 3 patients with generalized pustular psoriasis (GPP), 2 patients with pustular dermatosis and 1 patient with neutrophilic dermatosis (144). Case reports demonstrated the successful treatment of GPP patients carrying mutations in the *IL36RN* gene (144–147). Clinical trials to evaluate Anakinra as treatment for patients with AD or inflammatory pustular dermatoses were ongoing at the time of the preparation of this manuscript. Finally, IL-1Ra treatment might also be beneficial for chemical-induced skin irritation (102).

Overall, administration of recombinant IL-1Ra may thus represent an interesting therapeutic option for a variety of inflammatory skin diseases (Table 2). The fact that daily administration of Anakinra is necessary to control inflammatory manifestations due to its limited half-life can be considered as a potential limitation for long-term therapy. In contrast, antibodies, which exhibit a longer circulating half-life and biological activity, may represent a practical approach for the management of chronic diseases. Since IL-1α is highly expressed in the skin and IL-1β may also be involved in the inflammatory process, bispecific antibodies targeting both cytokines or anti-IL-1R1 antibodies may represent more suitable approaches than antibodies targeting either cytokine separately (**Figure 5**).

**Table 2:** IL-1 family antagonists in human inflammatory skin diseases.

**Cytokine**	**Human skin disease**	**Observation**	**Effect in mouse model**
IL-1Ra	DIRA syndrome	Associated with *IL1RN* loss of function mutations (140–142) Remission upon Anakinra treatment (140, 143)	Genetic background dependent spontanous skin inflammation in *Il1rn^−/−^* mice (148, 149) but without full DIRA picture
	GPP, pustular, and neutrophilic dermatoses	Successful treatment with Anakinra (144–147)	–
	Psoriasis	Association with *IL1RN* gene polymorphism (139)	Anti-inflammatory effect of IL-1Ra *in vivo* (94, 148)
	Allergic contact dermatitis	Association with *IL1RN* gene polymorphism (138)	–
	CHS	–	Anti-inflammatory effect of IL-1Ra *in vivo* (150)
	Delayed skin wound healing in diabetic individuals	–	Anti-inflammatory effect and improved wound healing *in vivo* (151)
IL-36Ra	DITRA syndrome, GPP and subtypes	Associated with *IL36RN* loss of function mutations (152–177)	No spontaneous skin phenotype in *Il1f5^−/−^* mice (178)
	Psoriasis	Anti-inflammatory effect of IL-36Ra in skin explants (179)	Anti-inflammatory effect of IL-36Ra *in vivo* (24, 118, 180, 181)
IL-37	Psoriatic arthritis	Association with *IL37* gene polymorphism (182)	–
	Psoriasis	Anti-inflammatory effect of IL-37 in cultured keratinocytes (183, 184)	Anti-inflammatory effect of IL-37 *in vivo* (183)
	Behçet's disease	Anti-inflammatory effect of IL-37 in skin explants (185)	–
	CHS	–	Anti-inflammatory effects of IL-37 *in vivo* (186)
IL-38	DIRA sydrome	175-kb deletion on chromosome 2q13 including *IL1F10* (140, 141, 187)	No spontaneous skin phenotype in *Il1f10^−/−^* mice (118)
	Psoriatic arthritis	Association with *IL1F10* gene polymorphism (182)	–
	Psoriasis	Anti-inflammatory effect of IL-38 in cultured keratinocytes (124)	Anti-inflammatory effect of IL-38 *in vivo* (124, 135)
	Skin lesions in SLE	–	Anti-inflammatory effect of IL-38 *in vivo* (188)

*Described roles of IL-1 family antagonists in human skin diseases and corresponding mouse models*.

#### IL-1Ra Function in Mouse Skin

The regulation and importance of the IL-1Ra/IL1 ratio in mouse skin has been evaluated in several studies.

Epidermal sheets from transgenic mice overexpressing IL-1α or sIL-1Ra in basal keratinocytes released more icIL-1Ra or IL-1α, respectively, demonstrating a counter-regulation of IL-1α and IL-1Ra expression (111).

Mice deficient in all four IL-1Ra isoforms spontaneously developed cutaneous inflammation resembling human psoriasis, with features such as thickened epidermis, parakeratosis, infiltration of inflammatory cells into dermis, and epidermis and formation of neutrophil-rich microabscesses beneath the SC (148). Of note, this inflammatory phenotype was observed only in a BALB/c, but not in a C57BL/6 genetic background (148). A different spontaneous phenotype with epidermal scaling and focal inflammatory skin lesions, as well as epidermal thickening and increased dermal infiltration of inflammatory leukocytes, has been described with lower incidence for mice deficient in all IL-1Ra isoforms on the DBA/1 background (149), indicating that background-related genes modify the susceptibility to skin inflammation. The apparition of the macroscopic skin phenotype was completely abolished when these IL-1Ra-deficient mice were crossed with transgenic mice overexpressing human icIL-1Ra1 (149).

The application of phorbol 13-myristate 12-acetate (PMA) to mouse skin, a mouse model for acute skin inflammation, resulted in epidermal hyperplasia (increase in number of cells in an organ or tissue), leukocyte infiltration, increased *Il1a* mRNA production in keratinocytes and elevated levels of the acute-phase protein serum amyloid A (SAA) in WT mice. PMA-treated human icIL-1Ra1 transgenic mice on the DBA/1 background showed similar epidermal thickening and comparable *Il1a* mRNA levels as WT mice, however inflammatory cell infiltration and the increase in serum SAA were partially abolished (149). Deficiency in all IL-1Ra isoforms did not aggravate epidermal thickening and dermal inflammatory cell infiltration upon PMA-application compared to WT mice (149).

Mice specifically lacking the icIL-1Ra1 isoform developed aggravated Aldara (5% IMQ)-induced skin inflammation, as demonstrated by increased ear thickness and increased mRNA levels of key pro-inflammatory cytokines (94). The severity of skin inflammation was controlled by icIL-1Ra1 released during Aldara (5% IMQ)-induced lytic keratinocyte death. Furthermore, keratinocyte-derived icIL-1Ra1 was shown to be the main IL-1Ra isoform regulating Aldara (5% IMQ)-induced skin inflammation, because conditional knockout mice lacking all IL-1Ra isoforms in skin-infiltrating myeloid cells, displayed the same phenotype as WT mice (94). Finally, injection of neutralizing anti-IL-1α antibodies attenuated the Aldara (5% IMQ)-induced ear thickening in icIL-1Ra1-deficient mice, identifying icIL-1Ra1 as an antagonist for the alarmin IL-1α (94).

Furthermore, IL-1 plays an important role in a mouse model of contact hypersensitivity (CHS) induced by the hapten dinitrofluorobenzene (DNFB) (189). The local intradermal injection of recombinant human sIL-1Ra before DNFB challenge of sensitized BALB/c mice reduced ear swelling, inflammatory cell infiltration and edema in the dermis as compared to control mice. The local intradermal administration of sIL-1Ra to naïve BALB/c mice 5 h before sensitization also suppressed CHS, indicating an inhibitory role for IL-1Ra during both sensitization and elicitation of CHS (150).

Dysregulated inflammation also contributes to delayed skin wound healing in diabetic individuals. Injection of the drug Anakinra, the recombinant human soluble IL-1Ra isoform, into wound margins of diabetic db/db mice improved wound healing and reduced neutrophil and macrophage infiltration, compared to vehicle-treated wounds (151).

Cancer patients receiving epidermal growth factor antibody therapy often experience acneiform skin rashes (dermatoses characterized by papules and pustules resembling acne vulgaris) with neutrophil infiltration as adverse side effect. In a mouse model for this pathology, Anakinra administration reduced neutrophilic infiltrates in the skin (190).

Overall, these findings demonstrate an anti-inflammatory role of IL-1Ra in mouse models of skin inflammation. These studies further confirm the importance of the IL-1Ra/IL-1 balance in the control of skin inflammation in mice, at steady state and in response to pro-inflammatory triggers (Table 2).

### IL-36Ra

#### IL-36Ra Expression, Activity, and Signaling

The *IL36RN* (*FIL1, FIL1D, IL1F5, IL1L1, PSORP, IL1HY1, IL1RP3, PSORS14, FIL1DELTA*) gene [gene ID: 26525, human (*IL36RN*); 54450, mouse (*Il1f5*)] contains 4 coding exons and 2 alternative non-coding exons (114, 119), likely transcribed from at least 2 promoters (120). The protein encoded by the *IL36RN* gene presents around 50% homology with IL-1Ra (114, 115, 119, 120, 125, 134, 191–194), and the *IL36RN* and *IL1RN* genes share the same exon/intron organization, suggesting that they might have been duplicated from the same ancestor gene (192).

The IL-36Ra protein is composed of 12 β-strands and 11 connecting loops, and its β-trefoil fold structure and hydrophobic core are well-conserved with other IL-1 family members (191). IL-36Ra contains no conventional leader peptide sequence (114, 116, 119, 120, 125, 193) and is not secreted through the classical ER-Golgi pathway. However, the IL-36Ra protein can be recovered in supernatants of IL-36Ra overexpressing cells (114, 116, 125), suggesting that it can be secreted following alternative pathways, which remain to be identified. Additionally, it has been suggested that, like IL-1α (31), IL-36Ra could play an intracellular role (195).

Towne et al. demonstrated that artificially maintaining the presence of the initial methionine, which is normally removed by endogenous methionyl aminopeptidases, importantly inhibits the extracellular receptor antagonist activity of IL-36Ra, as compared to the naturally processed form starting at valine 2 (V2) (47). In addition, cleavage of a SUMO-TAG linked to the N-terminal part of IL-36Ra can be performed by neutrophil elastase *in vitro*, which also releases the V2 active form, suggesting that neutrophil elastase might complement methionyl aminopeptidases to produce the V2 active form (196). Of note, subsequent cleavage of the V2 form by neutrophil proteinase K or cathepsin G leads to formation of a truncated protein starting at serine 4, (S4) which no longer presents antagonistic properties (196).

*IL36RN* mRNA expression has been found in different tissues of mice and humans, but its expression in the skin is higher than in any other organ (103, 114–120, 125). In particular, *IL36RN* is highly expressed in mouse and human keratinocytes (103, 114–118), which constitute the most important IL-36Ra producers in the skin (116, 118). *IL36RN* is induced in differentiating keratinocytes destined to undergo cornification (a unique form of terminal differentiation and programmed cell death undergone by keratinocytes to form the SC), but its presence in cetaceans, which have lost the epidermal cornification program, suggests a role for IL-36Ra beyond induction of cornification (103). Human *IL36RN* is also constitutively expressed in other cell types including macrophages, monocytes, B cells or DCs (114, 115, 117, 119, 120).

AP-1, c-Fos, c-Jun, and NF-κB binding sites within the human *IL36RN* promoter region (197) suggest regulation by TLR ligands and cytokines. Indeed, TLR ligands such as LPS or poly I:C induce *IL36RN* expression in primary human keratinocytes (117), as well as in human THP-1 monocytic cells (119), but not in mouse bone marrow-derived DCs (121). Stimulation with TNF-α, IL-1β, IL-36α, IL-36β, IL-36γ or a combination of TNF-α, IL-1β, OSM, IL-17, and IL-22 additionally induced *IL36RN* expression in primary human keratinocytes (117, 195, 198).

IL-36Ra expression was increased in psoriatic skin in mouse models or patients (79, 116–118, 178, 180, 195, 199–204). IL-36 signaling in keratinocytes is mandatory for the development of Aldara (5% IMQ)-induced psoriasis (79, 205), suggesting that the observed increase in IL-36Ra is insufficient to counterbalance the effects of agonistic IL-36 cytokines. Indeed, *Il1f5* is strongly induced in skin of Aldara (5% IMQ)-treated mice (79, 195), and IL-36α- but not IL-36β- or IL-36γ-deficient mice are protected in this model (206). Another hypothesis could be related to the important, IL-36-mediated, neutrophil infiltration in psoriatic skin (24, 79, 205). Indeed, the supernatant of PMA-treated neutrophils has been shown to induce cleavage of IL-36Ra into the inactive S4 form (196). Thus, neutrophils, which also release enzymes activating agonistic IL-36 cytokines (199), might inhibit IL-36Ra activity by inducing cleavage into its inactive form, shifting the IL-36α/IL-36Ra ratio even more in favor of IL-36α.

In a murine model of AD induced by the vitamin D3 analog MC903, *Il1f5* expression was increased in treated skin at early time points, but decreased before the peak of disease (207). In allergic contact dermatitis, expression of all IL-36 cytokines was increased in involved skin or *ex vivo* skin explants from patients, except for IL-36Ra, suggesting that the lack of opposition to IL-36 signaling in these patients might drive inflammation (208). Finally, IL-36Ra expression was increased in keratinocytes of Kindler syndrome (a rare congenital disease that causes fragile and blistering skin) patients (209) and in tumors of skin cancer patients (197).

IL-36Ra acts as an antagonist of other IL-36 cytokines by binding specifically and competitively to IL-36R (Figure 2D) (47, 116, 121, 122, 210), with higher affinity than the IL-36 agonists (47, 123, 210). Because of the structure of the IL-36Ra β11/12 loop (123), binding of IL-36Ra to IL-36R prevents the recruitment of the co-receptor IL-1RAP, which is necessary to trigger subsequent signaling (47, 199, 210). The β4/5 loop also contributes significantly to the antagonistic properties of IL-36Ra (123).

Interestingly, several groups observed that, at high concentrations, IL-36Ra lost its antagonistic properties and presented either no or agonistic effects (122, 195, 211), suggesting that, at supra-physiologic concentrations, IL-36Ra might signal through IL-36R. This could be related to the recent observation that IL-36Ra binds with similar affinity to IL-36R alone or to the IL-36R/IL-1RAP heterodimer when heterodimerization is forced *in vitro* (210). In addition, in a model of brain inflammation, IL-36Ra induced production of IL-4 after interaction with SIGIRR, and thereby suppressed IL-1β- and LPS-mediated inflammation (212). Although this observation is out of the context of skin inflammation, it still suggests that the mode of action of IL-36Ra can be different from only competitive inhibition of IL-36 signaling. Moreover, SIGIRR is expressed by Th17 cells and regulates IL-17-induced EAE development in mice (213), indicating that IL-36Ra might exert direct inhibition of Th17 cells present in psoriatic environment. This hypothesis still requires further confirmation.

In summary, IL-36Ra is expressed in keratinocytes and immune cells of the skin (Table 1) and its expression is enhanced in the context of skin inflammation. Its main function so far characterized is the competitive inhibition of IL-36 signaling (Table 1, **Figure 5**). Nevertheless, several questions remain unsolved regarding the secretion of IL-36Ra, the regulation of its activity by proteases, its putative role in differentiating keratinocytes, or the function it might exert by direct signaling on SIGIRR^+^ Th17 cells in the context of skin inflammation.

#### IL-36Ra in Human Inflammatory Skin Diseases

GPP is a rare and severe subtype of psoriasis vulgaris (PV), which can be life threatening. It is characterized by fever and generalized rash with disseminated pustules throughout the body. Despite being clinically distinct from PV, GPP is associated with PV in around 30% of cases (152).

In 2011, 2 independent studies identified mutations of the *IL36RN* gene, coding for proteins with predicted functional defects, in patients with GPP (153, 154). Since then at least 25 mutations in the *IL36RN* gene have been identified in patients throughout the world and associated with all GPP subtypes (146, 152–176, 214–218), geographic tongue (inflammatory condition of the tongue) (219), impetigo herpetiformis (a form of GPP occurring in pregnancy) (160), acute generalized exanthematous pustulosis (a severe drug-induced dermatosis) (152, 153, 164), acrodermatitis continua of Hallopeau (a rare variant of pustular psoriasis) (160, 167, 169, 219), inverse psoriasis (a form of psoriasis that affects skin folds) (169) and palmoplantar psoriasis (a form of psoriasis affecting the skin of the palms and soles) (216). Most of the time, these mutations were predicted to interfere with IL-36Ra function using bioinformatic tools, and associated with disease severity (152). *In vitro* assays demonstrated that they either prevented IL-36Ra expression, or led to alteration of the function of the protein (152–162, 174, 177, 218, 219). Interestingly, expression of an inactive form of IL-36Ra was found in patients harboring a c.4G>T p.V2F mutation, which retained the initiator methionine, indicating that full-length IL-36Ra is indeed inactive [(155), see above]. The disease condition observed in patients with loss of function mutations of the *IL36RN* gene is referred to as DITRA (Deficiency for Interleukin Thirty-six Receptor Antagonist). DITRA patients represent a minor group among GPP patients, but have enhanced clinical severity and morbidity compared to other patients without mutations of *IL36RN* gene (152). Only 1 patient out of 10 in a cohort of sporadic GPP cases presented *IL36RN* mutations, suggesting that these variants are mostly found in familial GPP (173).

Mechanistically, the phenotypes observed in GPP patients correlate with dysregulated IL-36Ra function. Increased IL-36α expression was observed in keratinocytes of GPP patients (170, 215, 220), a phenotype that could be reversed by addition of recombinant IL-36Ra (215). Highly proliferating CD4^+^ T cells with restricted TCR repertoires were found in skin and blood of GPP patients, and were major producers of IL-17 in the skin. Importantly, these cells expressed IL-36R, and stimulation with IL-36 enhanced their TCR-mediated proliferation (175). IL-36 signaling also potentiated TLR9-induced IFN-α production by pDCs and a prominent type I IFN signature was associated with IL-36 deregulation in GPP and PV patients (214). However, only 4 out of 14 patients in this study presented *IL36RN* mutations, indicating that dysregulated IL-36 signaling may occur also in absence of *IL36RN* mutations. Indeed, IL-36 and IL-36Ra over-expression has been observed also in lesional skin of PV patients (116, 178, 195, 203, 204), in whom *IL36RN* mutations are not enriched (221).

Consistent with the presence of common disease mechanisms in patients with or without *IL36RN* mutations, classical psoriasis treatments, including granulocyte and monocyte adsorption apheresis (a procedure in which one or more blood components are removed to treat a disease) (158, 222), secukinumab (anti-IL-17A) (171, 217), anakinra (antagonist of IL-1R1) (145, 176), ustekinumab (anti-IL-12/IL-23) (175), or infliximab (anti-TNFα) (168) have been used successfully in patients carrying *IL36RN* variants.

Since DITRA patients present altered function or expression of IL-36Ra and dysregulated IL-36 activity, use of either blocking antibodies targeting IL-36R or recombinant IL-36Ra have been considered for therapy, with promising results. Inflammation in human lesional psoriatic skin transferred onto SCID mice is reduced by treatment with an anti-human IL-36R blocking antibody (223). In addition, recombinant IL-36Ra reduces the expression of psoriatic pro-inflammatory genes and the number of CD3^+^ T cells and CD11c^+^ DCs in *ex vivo* cultures of psoriatic skin, suggesting an effect of IL-36Ra on proliferation and/or survival of these cells (179). Most importantly, a recent clinical trial showed that anti-IL-36R treatment was effective in 7 GPP patients, with or without *IL36RN* mutations (224). Thus, targeting IL-36 signaling in GPP patients is a promising therapeutic strategy. Future head-to-head clinical trials comparing IL-36R signaling blockade with other therapies, including anti-IL-1 or anti-IL-17A antibodies, may provide interesting information on the specific role of IL-36 in the complexity of the cytokine network in patients with GPP with or without *IL36RN* mutations.

Overall, these studies highlight the important role of IL-36Ra in skin homeostasis and in the prevention of severe forms of psoriasis (**Figure 5**). Indeed loss-of-function mutations show strong association with GPP and IL-36Ra treatment attenuated the pro-inflammatory phenotype in keratinocytes from patients (Table 2). These discoveries led to the trial of anti-IL-36R antibodies as treatment for GPP, with promising results.

#### IL-36Ra Function in Mouse Skin

Since IL-36Ra is more importantly produced in the skin than in any other organ (103, 114–120, 125), and since IL-36 cytokines are involved in skin inflammation (199), it is expected that IL-36Ra exerts protective functions in inflammatory skin diseases. However, mice deficient in IL-36Ra do not present any spontaneous skin phenotype (178), indicating that removal of homeostatic IL-36Ra levels is not sufficient to trigger disease development. Nevertheless, this shift in the IL-36 agonist/antagonist ratio renders mice more sensitive to induction of skin diseases in several *in vivo* models (24, 118, 178, 181, 223, 225–230).

Indeed, IL-36Ra-deficient mice developed more severe Aldara (5% IMQ)-induced dermatitis than WT mice (24, 118, 181), with increased ear (24, 118) and epidermal thickness (24, 118, 181), increased dermal infiltrated area (118), and increased numbers of skin-infiltrating CD45^+^ leukocytes and more specifically neutrophils (24). However, no difference was observed between WT and *Il1f5*^−/−^ mice in terms of trans-epidermal water loss or expression of the Aldara (5% IMQ)-induced pro-inflammatory genes *Il36a, Tnfa, Il1b, Cxcl1*, or *Cxcl2* (181).

In the murine model of AD induced by MC903, *Il1f5* expression was transiently increased at early time points, but there was no difference in disease severity between WT and *Il1f5*^−/−^ mice (207). This result might be explained by the fact that *Il1f5* was only induced until day 3 of treatment, while the peak of the disease was observed after 9 days, a time when *Il1f5* expression had returned to baseline and when expression of IL-36 agonists was importantly induced, suggesting that the agonist/antagonist ratio is already too importantly shifted toward the agonist side to be counterbalanced by IL-36Ra.

Finally, *Il1f5*^−/−^ mice are more sensitive than WT mice to skin inflammation induced by subcutaneous LPS injection (181). In this model, IL-36Ra-deficient mice presented more severe skin abscesses and erythema, and an increased skin severity index, as compared to WT mice. In addition, IL-36Ra-deficient mice showed higher skin levels of the neutrophil chemoattractant CXCL1 than WT mice (181).

Dysregulation of the IL-36 agonist/antagonist ratio by overexpression of IL-36 agonists also favors spontaneous development of skin inflammation. Indeed, mouse neonates with constitutive overexpression of IL-36α in keratin (KRT)14^+^ keratinocytes developed spontaneous skin disease with features specific of psoriasis, such as flaky skin, increased epidermal thickness, increased proliferation of basal keratinocytes and increased skin expression of *Il1f5, Il36a, Il36b, Il36g*, or *Il23a* (178). However, this inflammatory skin phenotype was transient, and disappeared 2–3 weeks after birth. This might be explained by a counterbalancing effect of IL-36Ra, since IL-36Ra-deficient KRT14/IL-36α transgenic mice presented high mortality in neonates and severe skin inflammation in the few surviving mice (178). Of note, KRT14/IL-36α transgenic mice with deletion of a single *Il1f5* allele did not show increased mortality, but still presented spontaneous inflammatory skin symptoms, which did not resolve at 2–3 weeks of age (178). Thus, a strong shift in the IL-36 agonist/antagonist ratio is required to trigger spontaneous skin inflammation in mice.

Conversely, intra-dermal injection of recombinant IL-36Ra during PMA-induced skin inflammation in mice presenting constitutive activation of STAT3 in K5^+^ keratinocytes alleviated the symptoms (180). This indicates that shifting the balance toward a low agonist/antagonist ratio by injection of recombinant IL-36Ra might prove a useful therapeutic approach.

Taken together, studies in mouse models demonstrated that IL-36Ra plays a constant role in regulation of skin homeostasis. If IL-36Ra deficiency does not directly trigger the development of skin inflammation, its absence leads to increased sensitivity to stress-induced skin inflammation (Table 2). IL-36Ra shifts the ratio of agonist/antagonist IL-36 signaling below threshold levels required for initiation of a Th17 program and the development of psoriasis-like skin inflammation.

### IL-37

#### IL-37 Expression, Activity, and Signaling

The human gene encoding IL-37 (IL-1F7, FIL1, FIL1Z, IL-1H, IL-1H4, IL-1RP1, gene ID: 27178) is composed of 6 exons. Five alternatively spliced transcript variants, encoding five distinct IL-37 isoforms (IL-37a to IL-37e) have been reported [reviewed in Boraschi et al. (231)]. Exons 4, 5 and 6 encode 12 putative β-strands predicted to form the β-trefoil cytokine fold characteristic for IL-1 family members. IL-37 variants 1, 2 and 5 (IL-37b, IL-37d, and IL-37a) contain, respectively, exons 1 and 2, exon 1 or exon 3, spliced to exons 4, 5, and 6 and are believed to be functional cytokines. In contrast, variants 3 and 4 (IL-37c and IL-37e) are predicted to be non-functional due to abnormal folding, as they lack exon 4, which encodes part of the β-trefoil structure. IL-37b is likely the biologically most relevant *IL37* gene product, due to its wider expression pattern and higher abundance, and is often generically referred to as IL-37. Interestingly, there is no mouse ortholog to the human *IL37* gene.

Exons 1, 2 and 3 of the *IL37* gene appear to encode pro-peptide regions, which are possibly cleaved by intra- or extracellular proteases for IL-37 maturation. There is a predicted caspase-1 cleavage site in exon 1 and IL-37b can indeed be cleaved by caspase-1, as well as less efficiently by caspase-4 (38, 232). Putative cathepsin-K, elastase-2, and matrix metalloproteinase−9 cleavage sites are also predicted within the IL-37b pro-domain (233). IL-37 processing in keratinocytes or skin cells in general has not been specifically investigated.

Although the mechanisms of IL-37 release or secretion remain unresolved, precursor and/or mature forms of IL-37 were detected in culture supernatants of different cell types, including keratinocytes, and this release was increased in presence of extracellular ATP (38, 126, 232, 234).

In healthy individuals, *IL37* mRNA is expressed mainly in the skin, where keratinocytes, in particular in the SG, appear to be the major producing cell type (103, 125, 127). Based on this observation, IL-37 was suggested to play a role in keratinocyte differentiation and cornification.

Several studies showed reduced *IL37* mRNA expression in lesional psoriatic skin as compared to non-lesional and control skin (127, 203, 235, 236). One study further indicated that successful treatment of psoriasis patients with the Janus kinase (JAK) inhibitor tofacitinib restored *IL37* expression levels in the skin (237). Reduced IL-37 protein staining was also reported in keratinocytes of the SG in lesional psoriatic skin (127). However, another study described strong IL-37 protein expression in skin-infiltrated CD4^+^ T effector memory cells and in dermal macrophages in psoriatic lesions (183). *IL37* mRNA expression also appears to be decreased in AD (238, 239), although one study reported increased IL-37 protein expression in AD keratinocytes (240). Phototherapy increased *IL37* mRNA levels in AD skin (241). Finally, *IL37* mRNA levels were decreased in lesional skin in hidradenitis suppurativa (chronic inflammatory disease affecting apocrine gland-bearing skin) (242, 243). Taken together, these observations suggest that IL-37 expression is generally reduced during skin inflammation *in vivo*.

Cultured primary human keratinocytes express predominantly IL-37b (103, 126) and IL-37 expression levels markedly increased with cell differentiation *in vitro* (103), suggesting that the downregulation of IL-37 expression during skin inflammation *in vivo* may be related to keratinocyte de-differentiation. On the contrary, in proliferating cultured human keratinocytes, β-defensin-3-induced pro-inflammatory signaling rather increased IL-37b expression (126).

IL-37 signaling was mainly studied in human and mouse myeloid cells, using overexpression or full-length and N-terminally truncated recombinant forms of human IL-37b. These experiments yielded similar conclusions in both species, despite the fact that there is no natural ortholog for IL-37 in mice.

**Figure 4:**
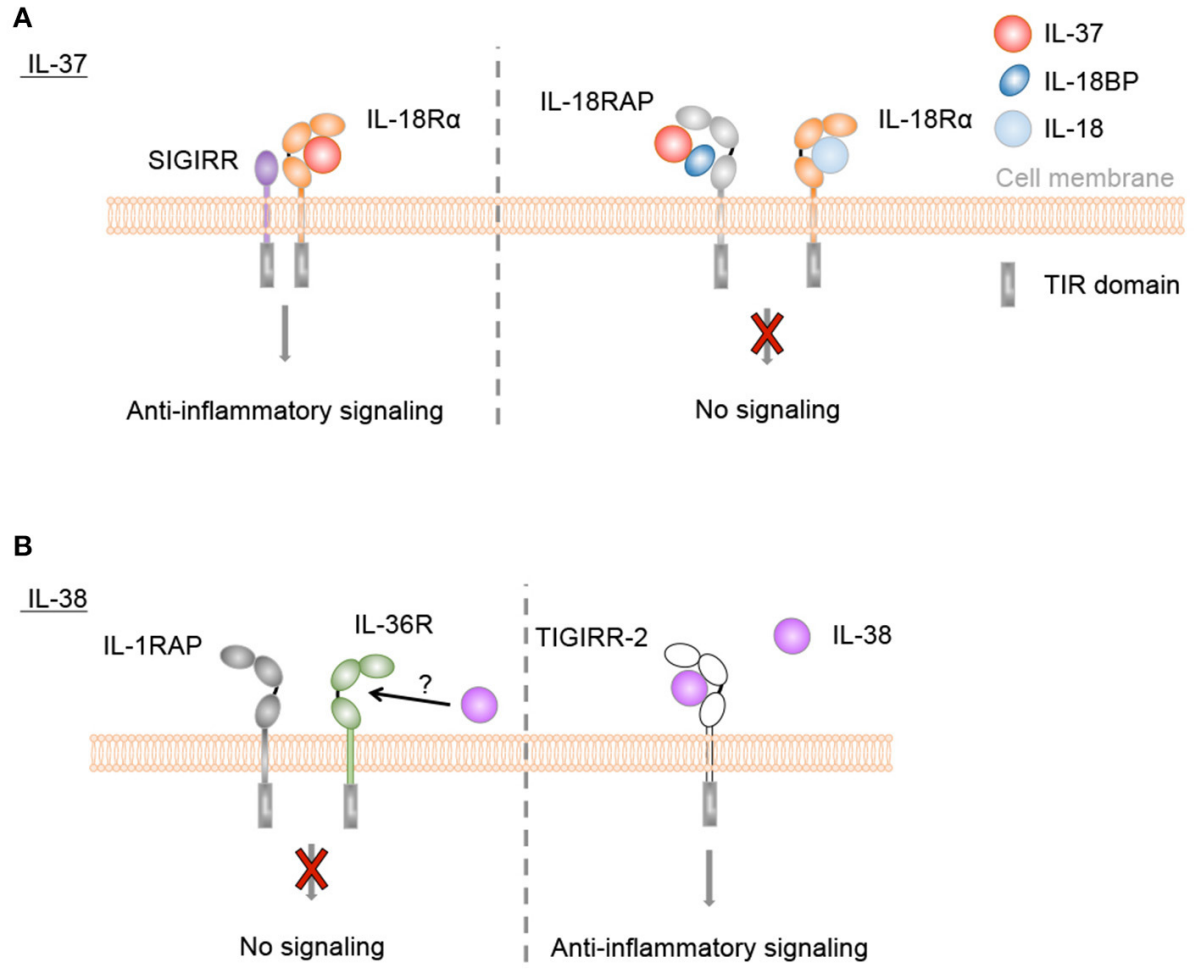
Anti-inflammatory IL-37 and IL-38 signaling. **(A)** IL-37 binds to IL-18Rα, but does not induce IL-18RAP recruitment. Instead, a complex of IL-18Rα and the inhibitory IL-1 family receptor SIGIRR is described to mediate anti-inflammatory effects of IL-37, such as inhibition of LPS or IL-1β-induced responses. Direct binding of IL-37 to IL-18BP and the formation of a heterotrimeric complex with IL-18RAP inhibits its association with IL-18Rα to transduce IL-18 signals. **(B)** IL-38 was reported to bind to IL-36R *in vitro* and to exert similar anti-inflammatory effects as IL-36Ra, although this has not been firmly demonstrated in *in vivo* studies. Furthermore, truncated IL-38 was proposed to limit inflammatory cytokine production by macrophages by acting as a ligand for TIGIRR-2.

Both precursor and processed IL-37b were described to bind to IL-18Rα (232, 234). Binding of mature IL-37b was more efficient than binding of the pro-form. However, the affinities of both forms were significantly lower than that of IL-18 (232). Association of IL-37 and IL-18Rα did not induce IL-18RAP recruitment or pro-inflammatory signaling. Instead, a complex of IL-18Rα and the inhibitory IL-1 family receptor SIGIRR was described to mediate anti-inflammatory effects of IL-37, such as inhibition of LPS or IL-1β-induced responses (Figure 4A). Many signaling pathways were modulated by IL-37, among which NF-kB, MAPK, mTOR, and inflammasome activation (128–132, 244, 245).

In addition, one study indicated that mature IL-37b enhanced the ability of IL-18BP to inhibit IL-18 activity at low IL-18BP concentrations (Figure 4A). This effect was proposed to rely on direct binding of IL-37b to IL-18BP and the formation of a heterotrimeric complex with IL-18RAP, which would inhibit its association with IL-18Ra to transduce IL-18 signals (133).

Furthermore, pro and mature forms of IL-37b form homodimers, although dimerization of the mature form is more efficient (232). It appears that the IL-37 monomer is the biologically active form of the cytokine. Dimerization at high IL-37 concentrations was therefore proposed to act as a negative feedback to avoid excessive immunosuppression (246).

Finally, while many studies point toward a broad inhibitory activity of IL-37 in innate inflammatory responses, effects of IL-37 on adaptive immunity, metabolism, angiogenesis, cell proliferation, and migration have also been reported (247–255).

IL-37 was proposed to act as a dual-function cytokine displaying intracellular anti-inflammatory effects in addition to its extracellular activity. In particular, intracellular IL-37b and IL-37d were described to interact with Smad3 and Smad3 inhibition or knockdown reversed anti-inflammatory effects of IL-37 in LPS or IL-1β-challenged cells or mice (37, 256). Further studies indicated that mature IL-37b translocates to the nucleus in a caspase-1-dependent manner and suggested nuclear anti-inflammatory effects for IL-37 (38, 257).

In summary, in healthy individuals, IL-37 is expressed mainly in the skin, where keratinocytes are the major producing cell type, and IL-37 expression is generally reduced during skin inflammation. Broad anti-inflammatory effects have been reported for extracellular IL-37, in particular in myeloid cells (Table 1). In addition, several reports suggest that IL-37 also exerts intracellular anti-inflammatory activity.

#### IL-37 in Human Inflammatory Skin Diseases

There is to date no known human syndrome linked to *IL37* loss or gain of function mutations. There are however a number of *IL37* genetic variants in the human population worldwide, some of which have been associated with inflammatory diseases, including psoriatic arthritis (182). Interestingly, several common and less common polymorphisms are non-synonymous mutations, leading to the production of variant proteins with variable anti-inflammatory potency (258–260).

Only few studies have addressed potential effects of IL-37 in the context of human skin inflammation, but their results concur to suggest anti-inflammatory activity of this cytokine. Indeed, overexpression of IL-37b in the human HaCat keratinocyte cell line decreased the expression of pro-inflammatory mediators, while, conversely, siRNA knockdown of *IL37* in HaCaT cells resulted in increased AMP expression (183, 184). Finally, *in vitro* treatment of inflammatory skin lesions of Behçet's disease patients with recombinant IL-37 also decreased cytokine expression (185).

**Figure 5:**
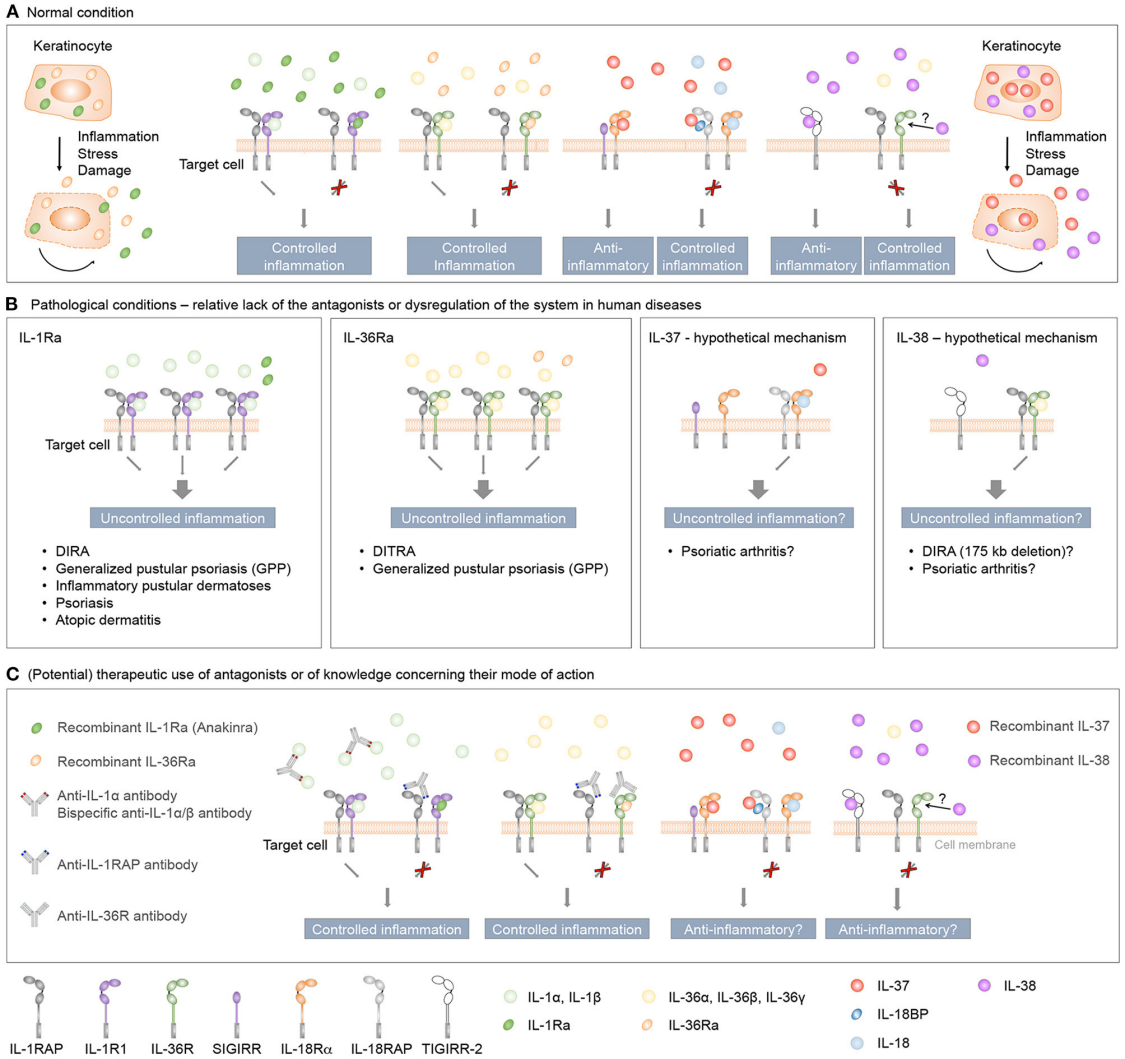
Role and therapeutic use of anti-inflammatory IL-1 family cytokines in human inflammatory skin diseases. **(A)** IL-1Ra, IL-36Ra, IL-37, and IL-38 are constitutively expressed in keratinocytes as intracellular proteins. During inflammation, or in response to stress or cell damage, these cytokines are passively released by dying cells or actively secreted through leaderless pathways, and exert regulatory roles to control skin inflammation. The classical receptor antagonists IL-1Ra and IL-36Ra specifically antagonize the effects of, respectively, IL-1 or IL-36 cytokines, while IL-37 and IL-38 exert broader anti-inflammatory effects. **(B)** Evidence derived from individuals with genetic deficiencies and clinical trials highlights essential roles for IL-1Ra and IL-36Ra in the regulation of the inflammatory response in human skin. While genetic association and *in vitro* studies also suggest anti-inflammatory properties for IL-37 and IL-38 in the context of human skin diseases, the role of these two cytokines in skin homeostasis *in vivo* remains to be determined. **(C)** Therapeutic agents developed to target IL-1 and IL-36 signaling include receptor antagonists and monoclonal antibodies against pro-inflammatory cytokines or their receptors. Since both IL-1R and IL-36R bind several agonists, bispecific antibodies neutralizing two agonists or antibodies blocking the receptors conceptually represent better therapeutic agents than antibodies specifically targeting a single ligand. A recently described monoclonal antibody targeting the co-receptor IL-1RAP may also prove useful to target IL-1 and IL-36 signaling simultaneously. Finally, it remains to be determined if treatment with recombinant IL-37 or IL-38 might be of therapeutic interest in specific inflammatory skin diseases.

Thus, genetic association and *in vitro* studies suggest that IL-37 might exert anti-inflammatory effects in human skin (Table 2). However, there is to date no known disease directly linked to loss of function or to reduced production of this cytokine. It thus remains to be determined if anti-inflammatory activity of IL-37 indeed contributes to human skin homeostasis *in vivo* or if treatment with recombinant IL-37 might be of therapeutic interest in specific inflammatory skin diseases (Figure 5).

#### Effect of IL-37 Treatment in Mouse Models of Skin Inflammation

IL-37b overexpression attenuated DNFB-induced skin CHS in mice by promoting the generation of tolerogenic DCs (186). Similarly, injection of a human IL-37b expression vector reduced disease severity, cytokine production and skin mast cell density in a keratin 14 VEGF-A-transgenic mouse model of psoriasis (183). Finally, a single intradermal injection of recombinant mature human IL-37b tended to reduce epidermal thickness, although it did not decrease inflammatory cytokine expression in a model of Aldara (5% IMQ)-induced skin inflammation (236).

Overall, the results of these studies suggest rather beneficial anti-inflammatory effects of IL-37 in mouse models of skin inflammation (Table 2). However, since mice lack a natural IL-37 ortholog, the significance of these observations remains uncertain.

### IL-38

#### IL-38 Expression, Activity, and Signaling

IL-38, encoded by the mouse *Il1f10* or human *IL1F10* [*IL1HY2, IL1-theta, FIL1-theta, FKSG75*, gene ID: 84639 (human), 215274 (mouse)] gene, is the least studied of the four IL-1 family members addressed by this review. *IL1F10* gene structure is very similar to that observed for all family members, displaying highly homologous regions within the last 3 exons (194). The gene comprises 4 exons and two transcript variants have been reported, each containing an open reading frame coding for an identical protein of 152 amino acids (aa). The IL-38 protein sequence shows its highest homology with the negative regulators IL-1Ra and IL-36Ra (39 and 43%, respectively, in human). Interestingly, evolutionary analyses suggested that *IL1F10* is a common ancestor of these 2 key modulators of innate signaling (261). Structurally, IL-38 displays the characteristic 12-β-stranded trefoil IL-1 fold, and some of the protein loops believed to be important for IL-1Ra and IL-36Ra function are largely conserved. Additional common structural features within the family have recently been extensively reviewed by Fields and colleagues (262). The *Il1f10* mouse ortholog is highly conserved and also encodes for a 152 aa protein with 81.6 % homology to human IL-38.

Early studies found *IL1F10* mRNA expression in human heart, placenta, fetal liver, spleen, thymus, tonsil, and skin (134). Additional analyses confirmed protein expression in all epidermal layers (124, 134–136) and in proliferating B cells of the tonsil (134). A more recent gene expression analysis pointed to the epidermis as the organ of the body with highest *IL1F10* expression (The GTEx Portal. Analysis Release V8. dbGaP Accession phs000424.v8.p2). In addition, *IL1F10* levels were potently induced in keratinocytes of skin equivalents and *in vitro* reconstructed human epidermis (RHE) presenting all differentiated epidermal layers as compared to subconfluent monolayers of cultured primary keratinocytes (103, 136).

IL-38 expression was also explored in psoriatic lesional and non-lesional skin, and compared to control skin. Similar *IL1F10* levels were observed in one study (235), while others showed IL-38 downregulation in psoriatic skin at the mRNA (117, 124, 127) and protein levels (124). Finally, one study reported increased *IL1F10* expression in PBMCs of psoriatic patients, which correlated with disease severity (263). In cultured normal human primary keratinocytes, *IL1F10* was constitutively expressed and significantly induced by a pro-inflammatory cytokine cocktail including IL-17A, IL-22, oncostatin M, IL-1β, and TNF-α (117). Conversely, another study showed reduced expression of IL-38 after stimulation of post-confluent primary keratinocytes with IL-17A, IL-22, IFN-γ, and IL-36γ, but not TNF-α (124). Most of these data point toward a decrease in IL-38 expression in psoriatic skin. The observation that there is a loss of epidermal differentiation in psoriatic processes (264) is *a priori* consistent with the notion that IL-38 expression is decreased in undifferentiated keratinocytes (75). Current data also suggest a relation between IL-38 and Th17 cytokines. However, the exact causes and consequences of changes in IL-38 expression in the pathophysiology of psoriasis remain to be fully understood.

Unlike in psoriatic skin, IL-38 was overexpressed in both perilesional and lesional skin of hidradenitis suppurativa patients (265). Finally, one study reported increased IL-38 levels in the serum of a set of systemic lupus erythematosous (SLE) patients compared to controls. However, the protein was not detected in most of the individuals of the original cohort of the study (266). As epidermis is bound to be a major source of IL-38, additional studies focusing on SLE patients with skin implication could yield interesting information.

*Il1f10* is also constitutively expressed in healthy mouse epidermis, and significantly decreased during Aldara (5% IMQ)-induced skin inflammation (117, 118, 135). Cultured primary mouse keratinocytes isolated from naïve WT BALB/c mice showed constitutive *Il1f10* expression, which was further induced upon IL-36β stimulation (118).

Extracellular functions for IL-38 were first described in 2012. Similar to IL-36Ra, IL-38 reduced production of *Candida albicans*-induced Th17 cytokines, and IL-36γ-induced IL-8 by PBMCs. Further *in vitro* immobilized receptor binding assays suggested the interaction of IL-38 with IL-36R (Figure 4B), however this has not been firmly demonstrated in *in vivo* studies. Interestingly, whereas IL-1Ra consistently and dose-dependently inhibited *C. albicans*-induced IL-22 secretion by PBMCs, the effects of IL-38 and IL-36Ra decreased at higher concentrations (122). Similar bell-shaped dose dependencies have been observed in several studies for the effects of recombinant IL-38 proteins (50, 124, 135). IL-38 released by apoptotic cells also inhibited IL-6 and IL-8 production by macrophages and reduced their ability to promote IL-17 production by human T cells (50). *In vitro* binding assays suggested that this effect was mediated by TIGIRR-2 (50) (Figure 4B), as recently further supported by data obtained in a TIGIRR-2-deficient mouse model (135). Overexpression of IL-38 in PMA-differentiated THP-1 cells reduced the secretion of IL-6, TNF-α, IL-23, and IL-10. Further analyses revealed the presence of IL-38 protein in the supernatant of the transduced THP-1 cells, and this supernatant reduced LPS-induced secretion of IL-6, TNF-α, and IL-23, but not IL-1β, by the parental THP-1 line, IL-6, and IL-23 secretion by LPS-stimulated M1 macrophages of healthy donors and IL-1β-induced IL-6 production by synovial fibroblasts of rheumatoid arthritis patients, suggesting an extracellular effect of the cytokine (137). Finally, in human keratinocytes, IL-38 antagonized the activation of p38 and NF-κB pathways by IL-36γ, leading to a reduction in the expression of pro-inflammatory markers (124).

Interestingly, two forms of IL-38 with different molecular weight were detected in both viable and apoptotic tumor cells. Further investigation of the apoptosis-derived IL-38 polypeptides revealed processing of full-length (FL) IL-38 into several N-terminally truncated forms. The shortest form (aa 20-152) reduced IL-1β-induced IL-6 production by human macrophages *in vitro*, while the FL form showed opposite effects. Recombinant FL IL-38 further enhanced LPS-induced IL-6 production by macrophages (50) and DCs (122), suggesting a context-dependent pro-inflammatory role of FL IL-38. In contrast, the production of IL-6 by LPS-stimulated macrophages was unaltered by truncated aa 20-152 IL-38 (50).

Collectively, the role of IL-38 in inflammation thus remains to be fully clarified. Although, in skin, IL-38 seems to be predominantly anti-inflammatory (Table 1), the different effects reviewed suggest that results depend on the cell type, stimuli and concentration of the cytokine. Whether IL-38 is able to self-associate to modulate its activity, as observed for IL-37 (233, 246), is still unknown. In addition, N-terminal protein truncation seems to alter IL-38 function. Finally, mouse *in vivo* data support the role of the TIGIRR-2 receptor in IL-38 signaling and similar *in vivo* approaches could shed light on the participation of other receptors.

#### IL-38 in Human Inflammatory Skin Diseases

There is no known human syndrome specifically linked to *IL1F10* loss or gain of function mutations. However, as previously mentioned, patients carrying a 175 kb deletion on chromosome 2q, encompassing the genes coding for IL-36γ, IL-36α, IL-36β, IL-36Ra, IL-38, and IL-1Ra, suffer from a severe autoinflammatory syndrome, classified as DIRA. In some of these patients, Anakinra treatment induced complete clinical remission, while others showed an incomplete response, suggesting that in addition to *IL1RN* other deleted genes, including *IL1F10*, might contribute to the observed phenotype (140, 141, 187). An IL1F10 gene polymorphism has also been associated with psoriatic arthritis (182).

Mercurio et al. showed that IL-38 upregulates, in dose-dependent manner, the production of differentiation markers such as KRT1, KRT10 and loricrin, but not the proliferation marker ΔNp63 in primary keratinocytes. During the inflammatory response, treatment of primary keratinocytes with FL IL-38 recombinant protein decreased IL-36γ-induced cytokine, chemokine and AMP mRNA levels (124).

Although genetic association and *in vitro* experiments suggest a role for IL-38 in the regulation of human skin inflammation (Table 2), more studies are needed to fully understand the functions and mechanism of action of IL-38 and thus, its potential involvement in inflammatory skin diseases (Figure 5).

#### IL-38 Function in Mouse Skin

The role of endogenous IL-38 in mouse skin inflammation was investigated in the Aldara (5% IMQ) model. IL-38 knockout mice treated with Aldara (5% IMQ) on their back skin showed a delayed resolution of skin inflammation (135), while IL-38-deficiency had no impact on the development or the resolution of skin inflammation in mice treated on their ears and no significant differences in mRNA expression of pro-inflammatory mediators were detected in ears of IL-38-deficient, as compared to WT mice after Aldara (5% IMQ) treatment (118).

Studies in mouse models for inflammatory skin diseases globally showed anti-inflammatory activity of IL-38 treatment. Subcutaneous injections of IL-38 in the back skin of WT mice ameliorated the symptoms of Aldara (5% IMQ)-induced skin inflammation by decreasing acanthosis, scale thickness and dermal inflammatory infiltrate (124). The epidermis of those mice also showed a decrease in infiltrating CD3^+^ T-cells and Ly6G^+^ neutrophils, in VEGF-A and Ki67 protein expression and in *Il6, Cxcl8, Ccl20, Il36* and *Il1f5* mRNA levels (124). Interestingly, injections of IL-38 normalized the expression of KRT10 and restricted its localization to the suprabasal layers (124). Similarly, IL-38 knockout mice treated with Aldara (5% IMQ) on their back had a better disease recovery when injected with recombinant IL-38 protein (135). Consistently, IL-38 injections in the skin of IL-38-deficient mice reduced IL-17 and IL-6 production and increased KRT10 expression compared to control mice (135). Chu et al. showed a markedly reduced skin lesion severity and skin infiltrations after intravenous recombinant IL-38 application in Murphy Roths Large (MRL)/lpr mice, which spontaneously develop an SLE-like disease (188).

Although, as for IL-36Ra, IL-38 deficiency does not directly trigger the development of skin inflammation in the mouse, studies in models of inflammatory skin diseases generally showed an anti-inflammatory activity of IL-38 (Table 2).

## Discussion

There is strong evidence that members of the IL-1 cytokine and receptor family play important roles in inflammatory skin pathologies such as psoriasis or AD. This review summarized the up-to-date literature and the importance of the anti-inflammatory functions of the four IL-1 family cytokines IL-1Ra, IL-36Ra, IL-37, and IL-38 in skin inflammation.

All 4 members are constitutively and highly expressed at steady state in the skin, with keratinocytes being the major producing cell type. IL-1Ra and IL-36Ra thus exhibit the same tissue distribution as their pro-inflammatory agonists IL-1α (in mouse and human), IL-1β (only in human), IL-36α, IL-36β, and IL-36γ. This raises the question why the antagonists are expressed together with the agonists in a place where immediate inflammatory response has to take place as a first line defense mechanism of the body against pathogenic organisms or noxious environmental triggers. Do the four herein-described anti-inflammatory IL-1 family cytokines act as anti-alarmins, as suggested for the IL-1Ra isoform icIL-1Ra1, which counteracts the pro-inflammatory activity of keratinocyte-derived IL-1α in a mouse model for psoriasis (94)? Or is a further role of the anti-inflammatory IL-1 family cytokines to maintain skin tolerance and regulate the composition of the commensal skin microbiota, as demonstrated for IL-1Ra and intestinal tolerance (267)? Another possibility is that they might exert additional functions, which are not related to inflammation, for instance in skin physiology during keratinocyte differentiation and cornification, as suggested for IL-37 (103) or IL-38 (124).

There are several additional knowledge gaps. IL-1Ra for instance was first described in 1984 (268). Since then, its function was investigated in countless biochemical, structural and cell biological studies. However, most *in vitro* and *in vivo* studies both in mouse and human were restricted to the secreted sIL-1Ra isoform, whereas the intracellular isoforms icIL-1Ra1,2,3 have been neglected. Indeed, there is so far only one *in vivo* mouse study about icIL-1Ra1 demonstrating a beneficial effect of this isoform in skin inflammation, which was published in 2020 (94). It is still unclear why IL-1Ra, in contrast to the other antagonists, exists as 4 different isoforms. Also, sIL-1Ra and icIL-1Ra1 have similar biological activities (90), although their N-termini differ by 7 amino acids in length, which contrasts with the observation that the biological activity of IL-36Ra is strictly dependent upon precise N-terminal trimming to V2.

While the classical antagonists IL-1Ra and IL-36Ra are well-characterized and extensively-described proteins with a precise function, namely to antagonize the binding of IL-1 or IL-36 cytokines, respectively, to their respective receptors, broad anti-inflammatory functions have been described for the “new” anti-inflammatory cytokines IL-37 and IL-38. It is still not clear if IL-37 and IL-38 also exert a specific antagonistic role by binding to one particular receptor to block the inflammatory activity of a given agonist. Finally, the question about potential intracellular functions, especially for the icIL-1Ra isoforms and IL-37, for which such intracellular roles have been described *in vitro*, remains mostly unanswered. Additional biochemical, structural and biological studies are thus required in order to further characterize the novel cytokines IL-37 and IL-38, as well as the intracellular IL-1Ra isoforms.

The anti-inflammatory characteristics of IL-1Ra, IL-36Ra, IL-37, and IL-38, as well as their constitutive expression in keratinocytes, at the site of skin inflammation suggest that they could represent interesting therapeutic options for inflammatory skin diseases. In this context, most of the *in vivo* studies in mice and clinical trials in humans focused on psoriasis, although there is some evidence that the herein-described anti-inflammatory cytokines might also play a beneficial role in AD or allergic contact dermatitis. The fact that the organization of mouse and human skin is very different, also notably the absence of an *IL37* gene ortholog in mice, makes direct transfer of results obtained with mouse models difficult. Nevertheless, as illustrated by the human DIRA and DITRA syndromes, endogenous IL-1Ra and IL-36Ra clearly play important roles in skin homeostasis.

Although some pre-clinical observations indicate that IL-37 and IL-38 possess anti-inflammatory properties and may thus prove of potential value in modulating inflammatory responses, evidence derived from both clinical trials and individuals with genetic deficiencies has identified IL-1 and IL-36 as better therapeutic targets. Future studies aimed at a better identification of receptors and downstream molecular cascades induced by IL-37 and IL-38 will be necessary prior to the development of therapeutic strategies using or targeting these cytokines.

Patients with gain-of-function mutations or genetic deficiencies were extremely useful to define the role of IL-1 cytokines in some inflammatory skin disorders and provide important information for targeted therapies. However, targeting other cytokines than those specifically associated with a given genetic mutation has also proven to be effective. For example, patients with DITRA responded favorably to IL-1 inhibition, most likely due to the production of IL-1 downstream of excessive IL-36 signaling (146, 269). In contrast, the effect of IL-36 blockade in patients with excessive IL-1 signaling, such as in DIRA, has not been tested. However, despite the presence of skin inflammatory lesions, the clinical features are more widespread in these patients and it is doubtful that IL-36 blockade could be sufficient to interfere with the full spectrum of systemic DIRA manifestations.

Different therapeutic agents have been developed to target IL-1 and IL-36, including receptor antagonists, and monoclonal antibodies against the cytokines or their receptors. The use of recombinant IL-1Ra and IL-36Ra as therapeutic agents has the advantage of blocking the signaling activity induced by all the different agonists, including IL-1α and IL-1β for the former and IL-36α, IL-36β and IL-36γ for the latter. However, as mentioned above, these recombinant proteins have relatively short half-lives and thus need to be administered more frequently than monoclonal antibodies. Due to the possible concomitant involvement of more than one agonist in skin inflammation, antibodies blocking the receptors represent conceptually better therapeutic agents than antibodies against their ligands. In addition, a recently described monoclonal antibody with neutralizing activity on the co-receptor IL-1RAP may also prove to be extremely useful considering the pathogenic role of IL-1 and IL-36 in skin inflammation (270). Furthermore, the simultaneous blockade of IL-1, IL-33, and IL-36 using an anti-IL-1RAP antibody may also be of interest beyond already recognized indications such as DIRA, GPP, and DITRA, for other inflammatory skin diseases including psoriasis, AD, hidradenitis suppurativa, and pyoderma gangrenosum and pyogenic arthritis, pyoderma gangrenosum and acne (PAPA) syndrome.

In conclusion, the four IL-1 family cytokines IL-1Ra, IL-36Ra, IL-37, and IL-38 are constitutively expressed in keratinocytes. They exert regulatory roles in skin inflammation, and a better understanding of their biology might lead to novel therapeutic strategies for the treatment of human inflammatory skin diseases.

## Author Contributions

PM, JG, and GP wrote the manuscript. LM and AD-B contributed to writing and critically revised the manuscript. All authors contributed to the article and approved the submitted version.

## Conflict of Interest

The authors declare that the research was conducted in the absence of any commercial or financial relationships that could be construed as a potential conflict of interest.
